# Anti-Thymocyte Globulin Induces Neoangiogenesis and Preserves Cardiac Function after Experimental Myocardial Infarction

**DOI:** 10.1371/journal.pone.0052101

**Published:** 2012-12-20

**Authors:** Michael Lichtenauer, Michael Mildner, Gregor Werba, Lucian Beer, Konrad Hoetzenecker, Andrea Baumgartner, Matthias Hasun, Stefanie Nickl, Andreas Mitterbauer, Matthias Zimmermann, Mariann Gyöngyösi, Bruno Karl Podesser, Walter Klepetko, Hendrik Jan Ankersmit

**Affiliations:** 1 Clinic of Internal Medicine I, Department of Cardiology, University Hospital Jena, Jena, Germany; 2 Christian Doppler Laboratory for Cardiac and Thoracic Diagnosis and Regeneration, Vienna, Austria; 3 Department of Dermatology, Medical University Vienna, Vienna, Austria; 4 Department of Thoracic Surgery, Medical University Vienna, Vienna, Austria; 5 Ludwig Boltzmann Cluster for Cardiovascular Research, Vienna, Austria; 6 Department of Cardiology, Medical University Vienna, Vienna, Austria; Universityhospital Düsseldorf, Germany

## Abstract

**Rationale:**

Acute myocardial infarction (AMI) followed by ventricular remodeling is the major cause of congestive heart failure and death in western world countries.

**Objective:**

Of relevance are reports showing that infusion of apoptotic leucocytes or anti-lymphocyte serum after AMI reduces myocardial necrosis and preserves cardiac function. In order to corroborate this therapeutic mechanism, the utilization of an immunosuppressive agent with a comparable mechanism, such as anti-thymocyte globulin (ATG) was evaluated in this study.

**Methods and Results:**

AMI was induced in rats by ligation of the left anterior descending artery. Initially after the onset of ischemia, rabbit ATG (10 mg/rat) was injected intravenously. *In vitro* and *in vivo* experiments showed that ATG induced a pronounced release of pro-angiogenic and chemotactic factors. Moreover, paracrine factors released from ATG co-incubated cell cultures conferred a down-regulation of p53 in cardiac myocytes. Rats that were injected with ATG evidenced higher numbers of CD68+ macrophages in the ischemic myocardium. Animals injected with ATG evidenced less myocardial necrosis, showed a significant reduction of infarct dimension and an improvement of post-AMI remodeling after six weeks (infarct dimension 24.9% vs. 11.4%, p<0.01). Moreover, a higher vessel density in the peri-infarct region indicated a better collateralization in rats that were injected with ATG.

**Conclusions:**

These data indicate that ATG, a therapeutic agent successfully applied in clinical transplant immunology, triggered cardioprotective effects after AMI that salvaged ischemic myocardium by down-regulation of p53. This might have raised the resistance against apoptotic cell death during ischemia. The combination of these mechanisms seems to be causative for improved cardiac function and less ventricular remodeling after experimental AMI.

## Introduction

Even though advancements in pharmaceutical and interventional treatment have significantly reduced early mortality following acute myocardial infarction (AMI), ischemic cardiomyopathy developing after AMI still remains widely prevalent and represents an increasing economic burden in countries of the western world [1]. A decade ago, great hopes were put on the emerging field of stem cell therapy for preserving cardiac function in patients who have suffered an AMI. However, clinical trials showed only inconsistent short to midterm results or no benefit at all regarding long term effects of stem cell therapy in patients with AMI [2], [3], [4]. Studies confirmed that only a small proportion of transplanted cells remained in the ischemic myocardium and so the concept of stem cells forming new viable myocardium has been shattered by unsatisfactory results in large clinical trials. Viewed in this light, new mechanistic concepts were proposed such as the “The Dying Stem Cell Hypothesis” by Thum *et al.* [5], namely, that a large proportion of stem cells are already undergoing apoptosis at the time point of transfusion. The authors of this hypothesis argued that apoptotic cells are conferring immunomodulatory signals and thereby attenuating immunoactivation and excessive inflammation in the infarcted myocardium and by doing so circumventing ventricular remodeling. This hypothesis has been recently substantiated by work conducted by our group [6], [7] showing that injection of apoptotic peripheral blood mononuclear cells (PBMC) shortly after induction of AMI in a rat model significantly reduced infarction size and preserved ventricular function. Moreover, we showed that this protective effect seems not to be caused by the injected cells themselves but by paracrine factors secreted by these apoptotic cells during the process of programmed cell death. This mechanism was confirmed in both a small animal model of AMI and in a large animal model of ischemia reperfusion injury [8]. A comparable mechanistic principle was proposed by Földes *et al.* in 1970 [9]. In *in vivo* experiments they clearly showed that by injecting anti-lymphocyte serum shortly after AMI in rats (and thereby causing apoptosis in lymphocytes) the damage due to ischemia was significantly reduced in the myocardium. However, this promising therapeutic concept has not been further addressed until now.

Based on these underlying results we sought to investigate whether comparable beneficial effects could be induced using a pharmaceutical both providing a similar mode of action (i.e. inducing apoptosis in peripheral blood cells) and that has evidenced its clinical applicability in many trials over the last decades. For this purpose we have chosen to test the efficacy of rabbit derived anti-thymocyte globulin preparations in an experimental model of AMI.

ATG preparations have been used since the 1970s in allogeneic stem cell transplantation to prevent graft rejection and to attenuate graft-versus-host disease (GVHD) [10]. The therapeutic mechanisms induced by ATG are multifactorial and are not fully elucidated yet. Previous studies showed that multiple immunological effects are elicited by ATG, such as induction of apoptotic cell death in T cells [11], [12], depletion of T cells and antigen presenting cell (APC), keeping dendritic APC in immature tolerogenic state [13], interfering with mature dendritic APC functions [14] and induction of regulatory T (Treg) cells [15], [16], [17].

However, the clinical use of ATG preparations is sometimes limited by adverse reactions after infusion such as anaphylaxis caused by preformed anti-rabbit antibodies, thrombocytopenia, hypotension, fever and most prominently the cytokine release syndrome. This massive release of cytokines such as tumor necrosis factor alpha, interleukin-1 and interleukin-6 is caused by activation induced cell death (AICD) which is initiated after ATG antibodies binding to T cells. As cellular homeostasis within the infarcted myocardium is hinged on a very delicate equilibrium between overshooting inflammation due to cardiomyocyte necrosis or inadequate immunoactivation hindering removal of cell debris, initiation of scar formation and wound healing, it is not known yet whether administration of ATG in the context of myocardial ischemia might be advantageous to preserve cardiac function after AMI. We therefore sought to investigate the therapeutic effects of ATG administration in a rat model of AMI with a special emphasis on paracrine factors released from leucocytes after activation by ATG. Recent publications substantiated the hypothesis that paracrine factors secreted by activated or “stressed cells” could confer cytoprotective signaling via their secretome in manifold models of tissue ischemia [8] [18], [19] [20]–[21]. Other groups recently stated that ATG also shows beneficial effects in preserving tissue function in a primate model of peripheral ischemia due to reduced ischemia reperfusion injury [22]–[23]. Here in this study we provide evidence that ATG induces a massive release of pro- and anti-inflammatory cytokines but also of pro-angiogenic chemokines and growth factors and also triggers cytoprotective signaling in cardiac myocytes via down-regulation of p53. Moreover, intravenous administration of ATG induced neoangiogenesis and is effective in preventing myocardial damage and tissue remodeling in a rat model of AMI.

## Methods

### Cell Culture of Human PBMC

All experimental procedures were approved by the ethics committee of the Medical University Vienna (Ethikkommission der Medizinischen Universität Wien, Borschkegasse 8b/E06, 1090 Wien) and were conducted in compliance with the Declaration of Helsinki Principles. Human peripheral blood mononuclear cells (PBMC) were obtained from young healthy volunteers after informed written consent (amendment to the vote of the ethics committee of the Medical University Vienna #EK2010/034). Major inclusion criteria were: body mass index 18–28 kg/m2, no intake of anti-inflammatory drugs during the last two weeks, no acute infection during the last month and no chronic inflammatory disease. Cells were separated by Ficoll-Paque (GE Healthcare Bio-Sciences AB, Sweden) density gradient centrifugation as previously described. Cells were resuspended in serum-free UltraCulture Medium (Lonza, Switzerland) and cultured in a humidified atmosphere for 24 or 48 hours at a density of 2.5*10^6^ cells/mL, n = 8). For cell culture experiments, ATG (Thymoglobulin, Genzyme, Germany) concentration was 500 µg/ml as previous studies showed that at this concentration a maximum of apoptosis induction could be achieved [24]. Moreover, dose titration experiments were conducted investigating cytokine secretion in cell cultures of 2.5*10^6^ human PBMC incubated with increasing concentrations of ATG (10 µg, 50 µg, 200 µg and 500 µg).

### Whole Blood Culture Experiment

Heparinized whole blood was obtained from young healthy volunteers after informed written consent as described above (n = 8). For base values, whole blood was centrifuged 20 minutes after blood withdrawal and plasma was stored at −80°. Moreover, 20 µg and 100 µg rabbit ATG (Thymoglobulin, Genzyme, Germany) or intravenous immunoglobulin (IVIG; Intratect, Biotest, Frankfurt, Germany) were added per milliliter of whole blood from the same donors. Whole blood was incubated in a humidified atmosphere at 37°. After 24 hours ATG co-incubated whole blood was centrifuged and plasma was separated and stored at −80° until further evaluation.

### Verification of Induction of Apoptosis in Rat and Human PBMC

Rat PBMC for *in vitro* experiments were separated from whole blood obtained from prior heparinized rats by direct puncture of the heart. Cells were separated by Ficoll-Paque (GE Healthcare Bio-Sciences AB, Sweden) density gradient centrifugation. The obtained cells were resuspended in serum-free UltraCulture Medium (UltraCulture, Lonza, Switzerland) and rabbit ATG (100 µg/ml or 500 µg/ml, Genzyme, USA, cell concentration 2.5*10^6^) was added. The cells were cultured in a humidified atmosphere for 24 hours. Induction of apoptosis was measured by Annexin-V/propidium iodine (FITC/PI) co-staining (Becton Dickinson, Franklin Lakes, NJ, USA) on a flow cytometer.

Concomitantly, the same set of experiments was conducted using human PBMC separated from heparinized whole blood from healthy donors.

### ELISA Analysis

Supernatant levels of selected cytokines secreted by PBMC co-incubated with ATG were measured by utilizing commercially available enzyme-linked immunosorbent assay (ELISA, Duoset, R&D Systems, USA) kits for the quantification of TNF-alpha, IL-2, IFN-gamma, IL-1bata, IL-1ra, Il-4, IL-10, IL-8, GRO-α, ENA-78, MCP-1, VEGF, HGF and TGF-beta according to the manufacturer’s protocol. Optical density values were measured at 450 nanometer on an ELISA plate reader (Victor3 Multilabel plate reader, PerkinElmer, USA).

### Elispot Assay

Additively, an ELIspot assay investigating IL-10 release from human PBMC after co-incubation with ATG was conducted using a commercially available kit (R&D Systems, USA) according to the manufacturer’s instructions. Human PBMC from healthy donors were incubated at a cell concentration of 20000/cells per well (in a 96 well anti-IL10 pre-coated ELIspot membrane plate) with increasing concentrations of ATG (1 µg, 5 µg, 10 µg, 20 µg). Coloured spots were quantified on a light microscope.

### Quantitative Real Time Polymerase Chain Reaction (RT-PCR)

After RNA extraction from human PBMC incubated with 500 µg ATG or without (negative control) using RNeasy kit, (QiIAGEN, Vienna, Austria) following the manufacturer’s instruction, cDNAs were transcribed using the iScript cDNA synthesis kit (BioRad, Hercules, USA) as indicated in the instruction manual. mRNA expression was quantified by real time PCR with LightCycler Fast Start DNA Master SYBR Green I (Roche Applied Science, Penzberg, Germany) according to the manufacturer’s protocol. The primers for IL-8 (forward: 5′-CTCTTGGCAGCCTTCCTGATT-3′, reverse: 5′-TATGCACTGACATCTAAGTTCTTTAGCA-3′ ) and β-2-microglobulin (β2M, forward: 5′-GATGAGTATGCCTGCCGTGTG-3′, reverse: 5′-CAATCCAAATGCGGCATCT-3′) were designed as described previously [25]. The relative expression of the target genes was calculated by comparison to the house keeping gene β2M using a formula described by Pfaffl *et al.* [26]. The efficiencies of the primer pairs were determined as described [25].

### Human Cardiomyocyte Culture and Phospho-kinase Membrane Array

Primary human ventricular cardiac myocytes were obtained from CellSystems (CellSystems Biotechnologie, St. Katharinen, Germany) and were cultured in cardiac myocyte medium (CellSystems) at 37°C at a cell density of 3*10^5^ in 6-well plates for 24 hours. In order to evaluate effects transduced by ATG or via paracrine signaling, cardiomyocytes were co-incubated with ATG (500 µg/ml), with pooled conditioned cell culture supernatants obtained from 2.5*10^6^ human PBMC from healthy donors (n = 4) that were co-incubated with 500 µg for 24 hours (ATG SN) or without additive (control medium). After the incubation period of 24 hours, lysates of cardiomyocytes were prepared and were analyzed using a membrane array for the detection of phosphorylated signaling kinases (Human Phospho-Kinase Antibody Array, R&D Systems, USA) according to the manufacturer’s instructions.

### Experimental Rat Model of Acute Myocardial Infarction

Animal experiments were approved by the Committee for Animal Research, Medical University of Vienna (vote: BMBWK-66.009/0278-BrGT/2005). All experiments were performed in accordance to the Guide for the Care and Use of Laboratory Animals by the National Institutes of Health (NIH Publication No. 85–23, revised 1996). Acute myocardial infarction was induced in adult male Sprague-Dawley rats (weight 300–350 g) by ligating the left anterior descending artery (LAD). In short, animals were anaesthetized intraperitoneally with a mixture of xylazine (1 mg/100 g bodyweight) and ketamine (10 mg/100 g bodyweight) and ventilated mechanically. A left lateral thoracotomy was performed and a ligature using 6–0 prolene was placed around the LAD beneath the left atrium. Immediately after the onset of ischemia, 10 mg of ATG resuspended in 0.3 ml UltraCulture medium was injected in the femoral vein as a single dose. Injection of cell culture medium alone and sham operation served as controls in this experimental setting.

### *In vivo* Evaluation of ATG Induced Cytokine Secretion

After intraperitoneal anaesthesia with a mixture of xylazine (1 mg/100 g bodyweight) and ketamine (10 mg/100 g bodyweight) adult male Sprague-Dawley rats (n = 4 per group, weight 300–350 g) were injected intravenously with 10 mg of ATG into the femoral vein. No coronary artery ligation was performed in this set of experiments. After 16, 24 and 48 hours animals were sacrificed and blood samples were collected. Whole blood was centrifuged 20 minutes after blood withdrawal and plasma was stored at −80°. The samples of rat plasma were evaluated for the concentration of cytokines (CXCL-1/KC1, TNF-1alpha, IL-1beta and IL-10) using commercially available ELISA kits (ELISA, Duoset, R&D Systems, USA). For the measurement of SDF-1 concentration in rat plasma samples an ELISA kit obtained from BG Bluegene (Shanghai, China). Experiments were performed according to the manufacturer’s protocol. Optical density values were measured at 450 nanometer on an ELISA plate reader (Victor3 Multilabel plate reader, PerkinElmer, USA).

### Histology and Immunohistochemistry

Rats were sacrificed either 72 hours or 6 weeks after experimental infarction. Hearts were explanted and then sliced at three layers at the level of the largest extension of infarcted area (n = 4–5 for 72 hours analyses, n = 9–13 for 6 weeks analyses). Slices were fixed in 10% neutral buffered formalin and embedded in paraffin. The tissue samples were stained with hematoxylin-eosin (H&E) and Elastica van Gieson (EVG). Immunohistochemical evaluation 72 hours after AMI was performed using an antibody directed against CD68 (MCA 341R, AbD Serotec, Kidlington, UK). Tissue samples were evaluated on an Olympus AX70 microscope (Olympus Optical Co. Ltd., Tokyo, Japan) at 200× or 400× magnification and captured digitally using Meta Morph v4.5 Software (Molecular Devices, Sunnyvale, USA). Image J planimetry software (Rasband, W.S., Image J, U.S. National Institutes of Health, Bethesda, USA) was utilized to determine the area of necrosis after three days and the size of myocardial infarction after 6 weeks. Six weeks after induction of AMI planimetric evaluation was carried out on tissue specimens stained with EVG for better comparison of vital myocardium and fibrotic areas. Infarction size was expressed as a percentage of the total left ventricular area.

### Assessment of Cardiac Function by Echocardiography

Six weeks after induction of myocardial infarction, rats were anaesthetized with 100 mg/kg ketamine and 20 mg/kg xylazine. Echocardiographic examination was conducted on a Vivid 7 system (General Electric Medical Systems, Waukesha, USA). Analyses were performed by an experienced observer blinded to the treatment groups to which the animals were allocated. M-mode tracings were recorded from a parasternal short-axis view and functional systolic and diastolic parameters were obtained (ejection fraction, EF; shortening fraction, SF; left ventricular end-diastolic diameter, LVEDD; left ventricular end-systolic diameter, LVESD). Shortening fractional was calculated as follows: SF(%) = ((LVEDD – LVESD)/LVEDD)*100%.

### Statistical Methods

Statistical analysis was performed using GraphPad Prism software (GraphPad Software, La Jolla, USA). All data are given as mean ± standard error of the mean (SEM). The Wilcoxon-Mann-Whitney-Test and the Wilcoxon matched pairs test were utilized to calculate significances between the groups. The paired student’s t-test was utilized to calculate significances between cytokines in *in vivo* experiments evaluating ATG induced cytokine secretion. P-values <0.05 were considered statistically significant (p-values were expressed as follows: * p<0.05, ** p<0.01, *** p<0.001).

## Results

### Induction of Apoptosis in Human and Rat PBMC

Co-incubation of human PBMC with 500 µg ATG resulted in an induction of apoptosis as 78% of cells were positive for Annexin V after 24 hours. Counts of PI positive cells were below 5%. Baseline values showed 15% positivity for Annexin V for untreated cells. In rat PBMC the rate of apoptosis was lower compared to human cells as evidenced by an Annexin positivity of 53% after ATG treatment compared to 12% in untreated cell cultures (p<0.001, n = 4).

### Cell Culture Supernatants Obtained from PBMC Co-incubated with ATG Evidence a High Concentration of Cytokines and Chemokines

Previous studies showed that ATG not only leads to T-cell depletion but can also induce a kind of cytokine storm which is accompanied by a temporary increase of pro-inflammatory factors after intravenous administration. Here we sought to reconfirm this phenomenon *in vitro* and also tried to analyse a broader spectrum of paracrine mediators including cytokines, chemokines and growth factors. Cell cultures of human PBMC were supplemented with 500 µg/ml ATG. After 24 and 48 hours supernatants were collected and analysed by means of ELISA. As was to be expected a significant increase of TNF-alpha, IL-2 and IL-1beta was found. But to a much greater extent the secretion of pro-angiogenic chemokines such as IL-8, GRO-alpha, ENA-78 and MCP-1 was triggered by ATG. Moreover, also VEGF and anti-inflammatory mediators (IL-1ra, IL-10) were increased in cultures supplemented with ATG (Table 1).

**Table 1:** Induction of cytokine secretion by ATG in cell cultures of human PBMC.

Cell Culture						
	after 24 h			after 48 h		
	2.5*10^6^ PBMC	2.5*10^6^ PBMC+ATG	*sig.*	2.5*10^6^ PBMC	2.5*10^6^ PBMC+ATG	*sig.*
TNF-alpha	100.9±43.7	207.0±46.3	*	32.3±23.0	255.6±48.4	*
IL-2	0.0±0.0	91.2±25.9	‡	0.0±0.0	73.9±34.3	*ns*
IFN-gamma	0.0±0.0	77.8±27.2	*	0.0±0.0	140.4±41.7	*ns*
IL-1beta	0.0±0.0	707.0±185.7	‡	0.0±0.0	432.5±145.4	†
IL-1ra	2576.9±485.0	10050.2±859.4	‡	3853.6±966.5	13645.0±2338.8	†
IL-4	4.3±3.6	8.9±2.2	*ns*	6.3±2.9	21.1±5.1	†
IL-10	0.0±0.0	50.1±9.5	‡	0.0±0.0	168.9±33.6	†
IL-8	10656.8±1829.4	41343.3±677.5	‡	7977.2±1295.5	33955.0±5018.4	*
GRO-alpha	1568.9±582.5	38194.9±1265.5	‡	880.2±759.2	45698.3±1642.4	†
ENA-78	8860.5±2416.3	63909.5±1792.5	‡	7912.2±3062.5	79928.5±1828.1	†
MCP-1	5226.4±1352.5	26157.4±3159.3	‡	2245.5±1351.3	25338.4±1639.2	†
VEGF	63.2±29.8	135.9±61.8	*	84.7±25.4	254.8±31.4	†
HGF	96.2±20.9	139.3±22.4	*ns*	270.8±57.3	214.0±34.6	*ns*
TGF-beta	168.6±25.4	140.9±20.9	*ns*	137.2±39.8	215.3±48.7	*ns*

ATG showed a significant induction of pro- and anti-inflammatory cytokine secretion. Moreover, ATG also triggered the release of pro-angiogenic factors such as VEGF and CXC and CC chemokines (e.g. IL-8, GRO-alpha, ENA-78, MCP-1) in a time dependent fashion.

* p<0.05 vs. matched controls.

† p<0.01 vs. matched controls.

‡ p<0.001 vs. matched controls.

### Induction of Cytokine and Chemokine Secretion in Human Whole Blood

Heparinized whole blood obtained from healthy volunteers was supplemented with rabbit ATG at a dose that is comparable with ATG concentration in the peripheral circulation of patients after intravenous administration (i.e. 20 µg/ml). In a second group, 100 µg/ml ATG was added in order to observe dose dependent differences. Whole blood without additive and the addition of intravenous IgG immunoglobulin at the same concentration served as negative controls. After an incubation period of 24 hours samples were analyzed by means of ELISA. Pro-inflammatory factors previously described to increase after ATG administration such as IL-2, TNF-alpha, IL-1beta, IFN-gamma showed a dose dependent increase. To even greater extent anti-inflammatory mediators such as IL-1 receptor antagonist and IL-10 were found to be secreted after addition of ATG to whole blood cell cultures. Moreover, ATG also induced a strong increase in the release of pro-angiogenic chemokines such as IL-8, MCP-1 and GRO-alpha (Table 2).

**Table 2:** Accumulation of cytokines, chemokines and growth factors in whole blood.

Whole Blood Incubation								
	0 h	24 h	24 h +20 µg ATG	*sig.*	24 h +100 µg ATG	*sig.*	24 h +20 µg IVIG	*sig.*
TNF-alpha	0.8±0.5	11.9±9.7	69.9±15.3	*	58.8±17.6	‡	3.1±3.1	*ns*
IL-2	0.3±0.3	0.9±0.6	3.2±1.9	*ns*	36.4±8.8	‡	0.0±0.0	*ns*
IFN-gamma	8.7±8.7	39.9±22.6	715.0±84.9	‡	415.9±72.6	‡	2.6±2.6	*ns*
IL-1beta	1.4±0.6	39.5±8.7	52.8±6.6	*	171.7±27.9	‡	36.4±8.6	*ns*
IL-1ra	0.0±0.0	1030.8±204.8	20492.2±1935.6	‡	26902.0±2360.4	‡	916.3±226.1	*ns*
IL-4	21.8±5.6	25.6±7.6	25.7±6.2	*ns*	27.9±5.9	*ns*	17.7±3.2	*ns*
IL-10	1.3±0.7	1.7±0.6	17.2±4.4	‡	32.1±6.2	‡	4.8±1.9	*ns*
IL-8	202.7±7.3	2427.1±304.3	4801.2±1108.2	†	6478.3±1051.5	‡	2713.5±243.7	*ns*
GRO-alpha	5.8±1.5	130.7±31.0	126.3±32.4	*ns*	501.4±91.8	†	98.9±29.9	*ns*
ENA-78	59.6±34.2	1236.1±147.1	549.8±129.9	‡	1955.0±306.3	*ns*	1486.5±297.1	*ns*
MCP-1	0.0±0.0	57.2±27.3	1107.0±114.7	‡	1846.9±91.5	‡	98.4±35.7	*ns*
VEGF	137.6±26.2	126.9±23.6	88.1±16.0	†	74.3±14.9	†	62.6±19.6	*ns*
HGF	17.9±13.0	140.5±31.3	677.1±63.0	‡	567.4±63.8	‡	175.2±40.4	*ns*
TGF-beta	0.0±0.0	0.0±0.0	0.0±0.0	*ns*	0.0±0.0	*ns*	0.0±0.0	*ns*

ELISA analysis showed that ATG induced a significant dose dependent increase in the secretion of pro- and anti-inflammatory cytokines compared to untreated PBMC after 24 hours of culture. To an even greater extent the release of pro-angiogenic chemokines (e.g. IL-8, GRO-alpha, ENA-78, MCP-1) was triggered. The use of an IVIG preparation failed to initiate the secretion of the aforementioned factors.

* p<0.05 vs. 24 h.

† p<0.01 vs. 24 h.

‡ p<0.001 vs. 24 h.

### Up-regulation of the Pro-angiogenic Chemokine IL-8

**Figure 1:**
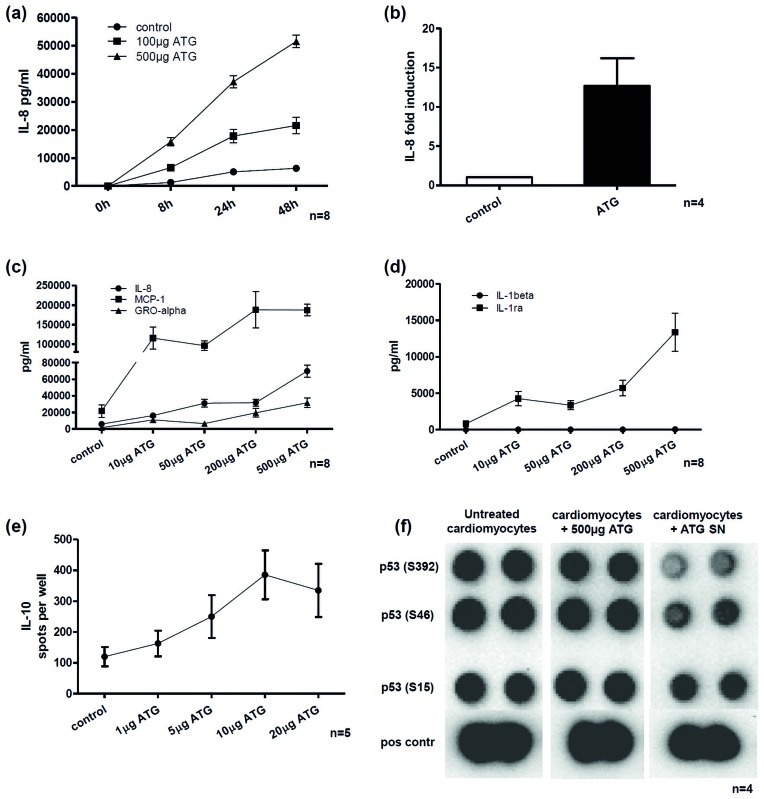
Effects induced by ATG on PBMC and cardiomyocytes. (a) ATG triggered a time dependent increase of IL-8 in cell cultures of PBMC. (b) ATG showed a 12.7 fold induction of IL-8 mRNA in human PBMC. (c) Co-incubating PBMC with increasing concentrations of ATG resulted in a dose dependent increase of chemokines in PBMC cultures. (d) The anti-inflammatory factor IL-1ra showed a much higher rate of induction by ATG compared to IL-1beta. (e) In an ELISpot assay co-incubation of PBMC resulted in a dose dependent enrichment of IL-10 producing cells. (f) Co-incubation of human cardiomyocytes with conditioned cell culture supernatants obtained from ATG stimulated PBMC (ATG SN) resulted in down-regulation of p53 whereas ATG itself shows no direct effects on cardiomyocytes.

As determined by ELISA, ATG induces a strong accumulation of IL-8 in cell culture supernatants and whole blood. In a time course experiment, IL-8 increased in a time and dose dependent fashion (Fig. 1a). In order to investigate whether this was solely caused by a passive release of IL-8 during the process of apoptotic cell death or via up-regulation of IL-8 synthesis, RT-PCR analysis was performed and showed a strong increase of Il-8 mRNA after ATG treatment (Fig. 1b).

### Dose Dependent Increase of Pro-angiogenic and Anti-inflammatory Factors in ELISA and ELISpot Assays

We further sought to determine whether ATG induced secretion of paracrine factors is dose dependent. After ATG was added to cell cultures of human PBMC a dose dependent increase of secretion was detected for pro-angiogenic chemokines (Fig. 1c). Also for IL-1beta a dose dependent effect was apparent, but the increase of IL-1ra exceeded that of IL-1beta 385-fold indicating an anti-inflammatory counter-response (Fig. 1d). In order to further validate this paracrine response induced by ATG we performed an ELISpot assay for IL-10. Adding ATG in increasing doses also resulted in a dose dependent release of IL-10 (Fig. 1e).

### Co-incubation of Human Cardiac Myocytes with Cell Culture Supernatants Obtained from ATG Treated PBMC Induces Down-regulation of p53

Based on these *in vitro* experiments indicating a strong paracrine response after treating PBMC with ATG we sought to investigate whether this has a direct or indirect effect on cardiac myocytes. Therefore primary human cardiomyocytes were incubated with 500 µg of ATG or with the conditioned cell culture supernatant derived from 2.5*10^6^ PBMC treated with 500 µg ATG (and thusly containing a broad mixture of cytokines, chemokines and growth factors as determined by ELISA). In order to gain an overview over regulatory pathways affected by ATG or by ATG conditioned supernatants a membrane array was performed. The most evident difference was found for p53 which showed a strong down-regulation after ATG conditioned supernatants were added to cardiomyocyte cell cultures. No differences were found when solely ATG was added (Fig. 1f, Figure S1, Table S1).

### *In vivo* Evaluation of ATG Induced Cytokine Secretion

**Figure 2:**
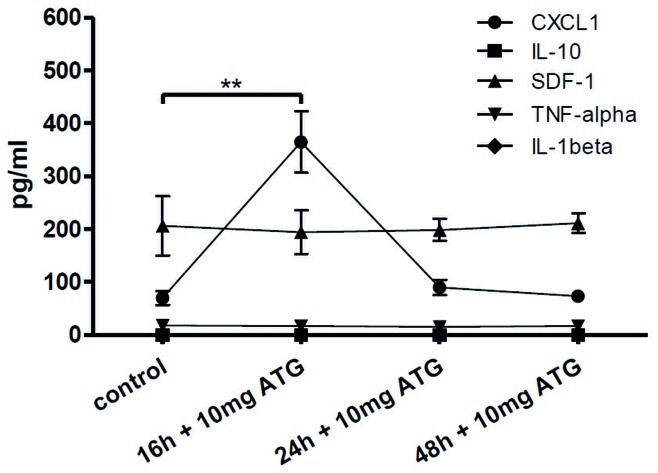
*In vivo* effects of ATG in an in vivo rat model. Sixteen hours after intravenous injection of 10 mg ATG CXCL1/KC1 showed a significant increase compared to baseline values. After 24 hours levels of CXCL1/KC-1 decreased again. TNF-alpha, IL-1beta, Il-10 and SDF-1 showed no differences after ATG injection.

In order to provide evidence that ATG also induced cytokine secretion *in vivo* rats were injected with 10 mg of ATG. Plasma samples were obtained after 16, 24 and 48 hours and evaluated by ELISA. The pro-angiogenic chemokine CXCL1/KC1 showed a significant increase 16 hours after ATG injection (364.7 pg/ml ±57.9 SEM, p<0.01, n = 4 per group, Fig. 2) and declined again after 24 h. No significant differences were observed for TNF-alpha, IL-1beta, IL-10 and SDF-1 (Fig. 2).

### Short Term Effects of ATG Treatment in a Rat Model of AMI Resulting in Less Myocardial Necrosis

**Figure 3:**
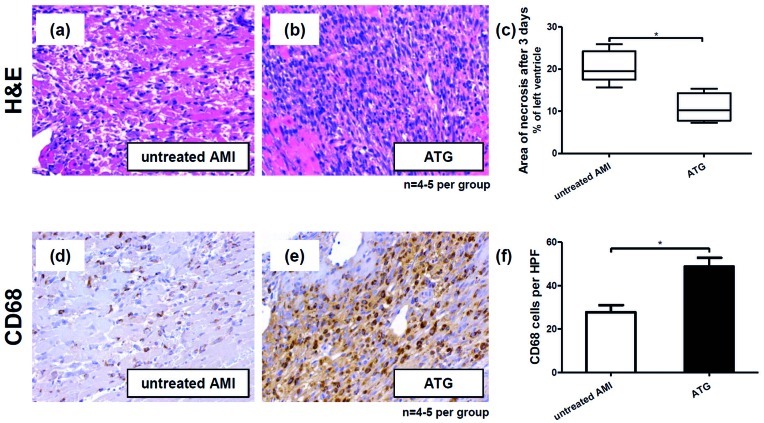
Beneficial short term effects of ATG in a rat AMI model three days after ligation of the left anterior descending artery. Compared to untreated controls (a) H&E stained specimens of infarcted myocardium from ATG treated rats showed much denser cellular infiltrates (b). The total infarcted area three days after induction of AMI was significantly reduced by ATG (c). Immuhistology showed an increased homing of CD68 positive monocytes/macrophages after ATG treatment (e,f) compared to controls (d).

Three days after ligation of the left anterior descending artery and induction of acute myocardial infarction rat hearts were explanted. In H&E stained tissue samples of infarcted myocardium from ATG treated animals showed a very dense cellular infiltrate compared to controls (Fig. 3a,b). In a planimetric analysis conducted on these tissue samples ATG treated rats evidenced a significant reduction of infarction size from 20.56% (±1.71 SEM) of the left ventricle to 10.77% (±1.71 SEM), (p<0.05, n = 4–5 per group, Fig. 3c). Immunohistological analyses were performed to further characterize the cellular infiltrate and showed that it was in large parts composed of CD68 positive cells (Fig. 3d,e). A quantification showed that in tissue samples from ATG treated rats a mean of 48.80 (±3.98 SEM) CD68 positive cells per high power field were found compared to just 27.80 (±3.22 SEM) in controls (p<0.05, Fig. 3f).

### Long Term Effects of ATG Treatment on Infarction Size, Ventricular Remodelling and Cardiac Function

**Figure 4:**
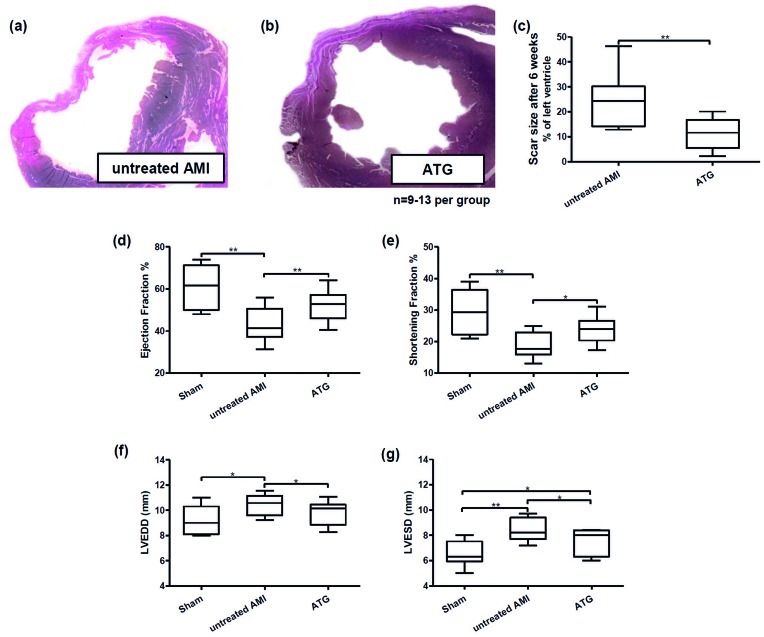
Beneficial long term results of ATG treatment six weeks after AMI. (a) Hearts obtained from untreated control animals evidenced an extensive scar size and signs of ventricular dilation, whereas scar size was reduced in ATG injected rats with a better preserved ventricular geometry (b). ATG injection after AMI resulted in a reduction of scar size from 24.95% of the left ventricle in untreated controls to 11.43% in the treatment group, p<0.01, n = 9–13 per group). Parameters of cardiac function (ejection fraction, shortening fraction, left ventricular end-diastolic diameter and left ventricular end-systolic diameter) were significantly improved by ATG (d,e,f,g).

Six weeks after induction of acute myocardial infarction hearts were explanted and analyzed microscopically. Hearts obtained from rats treated with ATG evidenced smaller scar areas and a better preserved ventricular geometry compared to untreated controls (Fig. 4a,b). A planimetric analysis showed a scar size of 24.95% (±3.59 SEM) of the left ventricle in control animals. In treated animals this was significantly reduced to 11.43% (±1.73 SEM, p<0.01, n = 9–13 per group, Fig. 4c). Moreover, an analysis of cardiac function conducted by echocardiography shortly before hearts were explanted showed a significant improvement of parameters in ATG treated rats compared to controls. Values for sham operated animals were the following: ejection fraction (EF) 60.47% ±3.70 SEM, shortening fraction (SF) 29.19% ±2.47 SEM, left ventricular end-diastolic diameter (LVEDD) 9.17 mm ±0.39 SEM, left ventricular end-systolic diameter (LVESD) 6.52 mm ±0.35 SEM. In untreated AMI control animals these parameters deteriorated to EF 42.91% ±2.22 SEM, SF 18.76% ±1.12 SEM, LVEDD 10.43 mm ±0.21 SEM, LVESD 8.47 mm ±0.23 SEM. Significantly improved values were recorded in ATG injected animals: EF 52.35% ±1.96 SEM, SF 23.86% ±1.11 SEM, LVEDD 9.81 mm ±0.25 SEM, LVESD 7.45 mm ±0.27 SEM (Fig. 4d,e,f,g, p-values between <0.01 and <0.05, n = 9–13 per group). Overall survival was better in ATG treated rats as AMI control animals showed a post-operative mortality rate of 40% whereas mortality of ATG injected rats showed a mortality of 23%.

### Increased Neoangiogenesis in the Borderzone between Viable Myocardium and Scar Tissue by ATG Treatment

**Figure 5:**
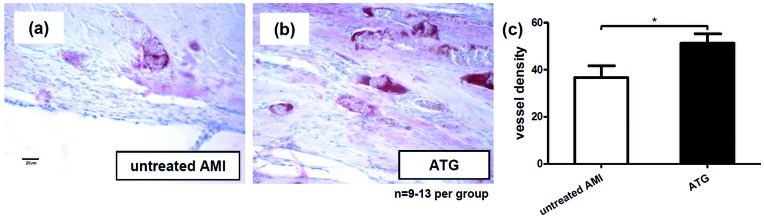
Enhanced neoangiogenesis by ATG after AMI. ATG treated animals showed a higher vessel density in the border zone between scar tissue and viable myocardium compared to untreated controls as shown in anti-vWF stained specimens (a,b,c).

Tissue samples of hearts explanted six weeks after induction of AMI were analysed by immunohistology using an antibody directed against van Willebrand factor to study neoangiogenesis the borderzone between collagenous scar tissue and viable myocardium. Hearts explanted from ATG treated animals evidenced a higher vessel density as compared to untreated controls. In controls a mean of 36.67±4.99 vessels were found, whereas by ATG injection vessel density was significantly increased to 51.25±4.02 (Fig. 5a,b,c, p<0.05, n = 9–13 per group).

## Discussion

Advances in treatment for patients with AMI, mainly by coronary intervention such as primary PCI, have led to a decrease in early mortality, but there remains a high incidence of subsequent heart failure among survivors. Here we present a new therapeutic concept using a pharmaceutical preparation that is successfully used in transplantation medicine for many decades. As known since the first studies that investigated ATG administration in patients these anti T cell antibodies can induce a prominent cytokine release. We confirmed this by experiments showing a strong increase of pro-inflammatory cytokine secretion such as TNF-alpha, IL-1 beta, IL-2 and IL-6 after co-incubation of PBMC with ATG. However, also anti-inflammatory factors such as IL-10 and IL-1ra showed a concomitant increase. To a much greater extent the release of pro-angiogenic chemokines such as IL-8, GRO-alpha, ENA-78 and MCP-1 was triggered by ATG. We also could show for the first time that VEGF secretion was significantly heightened by ATG. These results indicate a broad induction and release of paracrine factors that might be of relevance modulating the cellular response after an ischemic event. However, amending current medical treatment of AMI patients with the administration of single growth factors has failed to show any benefits [27]–[29]. On the contrary, reports indicate that a multifarious cocktail of paracrine factors could provide beneficial effects after AMI [8], [30]. Di Santo *et al.* showed that stressed endothelial progenitor cells showed a comparable secretory profile compared with our results [18]. They further provided evidence that by injecting this mix of cytokines, chemokines and growth factors neoangiogenesis and local perfusion was significantly improved in a hind limb ischemia model. Furhermore, Schuh *et al.* showed that injected of microspheres inducing a pro-inflammatory period after AMI led to reduction of infarction size and improvement of ventricular function [31]. They discussed that these beneficial effects seem to be mediated by a paracrine signaling of cytokines released during the early phase after AMI in the infarcted myocardium.

Here, we could show that administering a dose of 10 mg ATG intravenously induced the secretion of the pro-angiogenic chemokine CXCL-1/KC1. Moreover, ATG injection shortly after AMI significantly reduced myocardial damage after 3 days compared to untreated controls. This salvation of endangered myocardium seemed not very likely to be caused just by yet determined immunosuppressive effects of ATG influencing immunoreactions after AMI. We speculated that ATG could exert protective effects on cardiac myocytes, either directly or via a loop way over paracrine signaling. In an *in vitro* experiment we could show that the conditioned supernatant of ATG co-incubated cells (and thusly containing the broad mix of paracrine factors as shown in Table 1 and 2) induced a down-regulation of p53 in cultured human cardiomyocytes. Previous studies provided evidence that via a down-regulation of p53 cardiomyocytes could be more resistant to apoptotic cell death during ischemia [32], [33], [34], [35], [36], [37]. This experiment showed that this effect was mediated via paracrine signaling and was not directly conferred by ATG itself.

Immunohistological analyses further showed a strong enrichment of CD68 positive monocytes/macrophages in the infarcted area of the myocardium three days after induction of AMI. This finding nicely correlates with the massive release of MCP-1 induced by ATG leading to increased homing of CD68 positive cells into the myocardium. The changeover from the inflammatory phase after ischemia to the reparative phase is usually mediated by macrophages and thereby having a pivotal role in the healing process after AMI [38]–[39]. However, MCP-1 represents a largely dichotome factor in the context of myocardial infarction. In the literature both protective and aversive effects of MCP-1 after myocardial ischemia were discussed [40]–[42]. These aspects were extensively discussed in a review by Becker *et al.* [43]. Dewald *et al.* showed that via inhibition of MCP-1 both using antibodies and a knock-out model inflammation and ventricular remodeling was reduced after myocardial ischemia. However, MCP knock-out mice also evidenced a prolonged inflammatory phase and delayed replacement of injured cardiomyocytes with granulation tissue after myocardial infarction. The authors stated: “it is possible that in patients with acute myocardial infarction, delayed phagocytosis of injured cardiomyocytes may increase the arrhythmogenic potential or predispose to mechanical complications, such as rupture, or ventricular aneurysm formation” [40]. In murine models of acute myocardial infarction these complications are found very rarely. Translational studies are needed in large animal models of infarction to understand the real therapeutic potential and safety of targeting MCP-1 [43].

The administration of ATG in AMI also evidenced beneficial long term effects as the collagenous scar area was significantly reduced in ATG injected animal compared to untreated controls. Moreover, the ventricular geometry was better preserved in animals of the therapy group. These results correlated with improved parameters of cardiac function as rats that were injected with 10 mg of ATG evidenced ameliorated values for ejection fraction, shortening fraction and also for ventricular diameters. Compared with our previous studies investigating the cardioprotective effects of direct administration of supernatants obtained from apoptotic cells, ATG showed a comparable efficacy in this context. Though, absolute values of infarction size and parameters of cardiac function were slightly better when supernatants of apoptotic cells were administered in the same experimental setting [8]. This might be related to a “therapeutic window” and the fact that the induction and the release of paracrine factors by ATG takes a few hours to be truly effective whereas in our previous studies the conditioned supernatant of apoptotic cells was injected intravenously right after induction of AMI.

As ATG showed as strong induction of pro-angiogenic factors such as VEGF, IL-8 and other angiogenesis promoting chemokines we sought to determine whether this could also be demonstrated after AMI *in vivo*. Six weeks after induction of AMI a higher vessel density was found in the border zone between the collagenous scar tissue and viable myocardium in anti van Willebrand factor stained specimens obtained from rats injected with ATG.

Moreover, ATG with its known T cell depleting function shows many pleotropic effects that have not been studied in all detail so far. Lopez *et al.* discussed another important aspect of ATG functions showing an induction of T regulatory cells [15]. In our study we mainly focused on the effects on cytokine secretion and neo-angiogenesis. However, the aspect of immunomodulation by ATG should be addressed in further studies. Dobaczewski *et al.* showed in a CCR-5 knock-out model the secretion of cytokines after AMI was strongly induced, recruitment of T regulatory cells was impaired and ventricular remodeling was augmented [44]. Addressing these findings, ATG showed partly conflicting effects as a release of pro-inflammatory factors was detected in *in vitro* experiments. *In vivo* we observed a significant increase of CXCL1/KC1 sixteen hours after injection of 10 mg whereas pro-inflammatory cytokines (TNF-alpha, IL-1beta) showed no alterations. ATG could offer beneficial therapeutic properties in this context, as ATG induced effects should only last hours (cytokine secretion) to days (T cell depletion). In the days and weeks after AMI, ATG induced effects should ebb away and thereby should not directly affect ventricular remodeling in the chronic phase.

In conclusion, we provided evidence that ATG induced a massive release of not only yet know pro- and anti-inflammatory cytokines but also of pro-angiogenic chemokines and growth factors. This induction of paracrine signaling proved to be beneficial in a small animal model of AMI leading to increased neoangiogenesis after ischemia, improved parameters of cardiac function and to a reduction of infarction size.

### Limitations

As it is known that ATG is a pharmaceutical preparation with a narrow therapeutic window and serious side effects, the use in a clinical scenario such as AMI would require a profound knowledge of the mode of action and further research would be necessary in order to define exact therapeutic doses to reduce the risk of serious complications. In our study using a small animal model no serious adverse effects of ATG treatment were found, ATG injected animals even showed a reduced mortality rate compared to controls. Moreover, most of the *in vitro* experiments in this study have been carried out using human cells, whereas *in vivo* experiments investigated the use of commercially available ATG directed against human T cell antigens. Even though these antibodies usually show a high rate of cross reactivity and ATG also led to a profound induction of apoptosis in rat cells, the used concentrations of ATG in human and rat experiments cannot be compared directly as a loss of efficacy would be very likely between species.

## Supporting Information

Figure S1 **Membrane array analysis of intracellular signaling kinases affected by ATG conditioned PBMC supernatants in human cardiac myocytes.** As shown, p53 was down-regulated after exposure to conditioned supernatants obtained from ATG stimulated PBMC cultures.
(TIF)

Table S1 **Membrane array coordinates are shown for intracellular signaling kinases affected by ATG conditioned PBMC supernatants in human cardiac myocytes (see Figure S1).**
(DOC)
