# Supplementary figures and images for: Anti-Thymocyte Globulin Induces Neoangiogenesis and Preserves Cardiac Function after Experimental Myocardial Infarction
Source: PLoS One. 2012 Dec 20;7(12):e52101. doi: 10.1371/journal.pone.0052101 (PMC3527351; doi:10.1371/journal.pone.0052101)

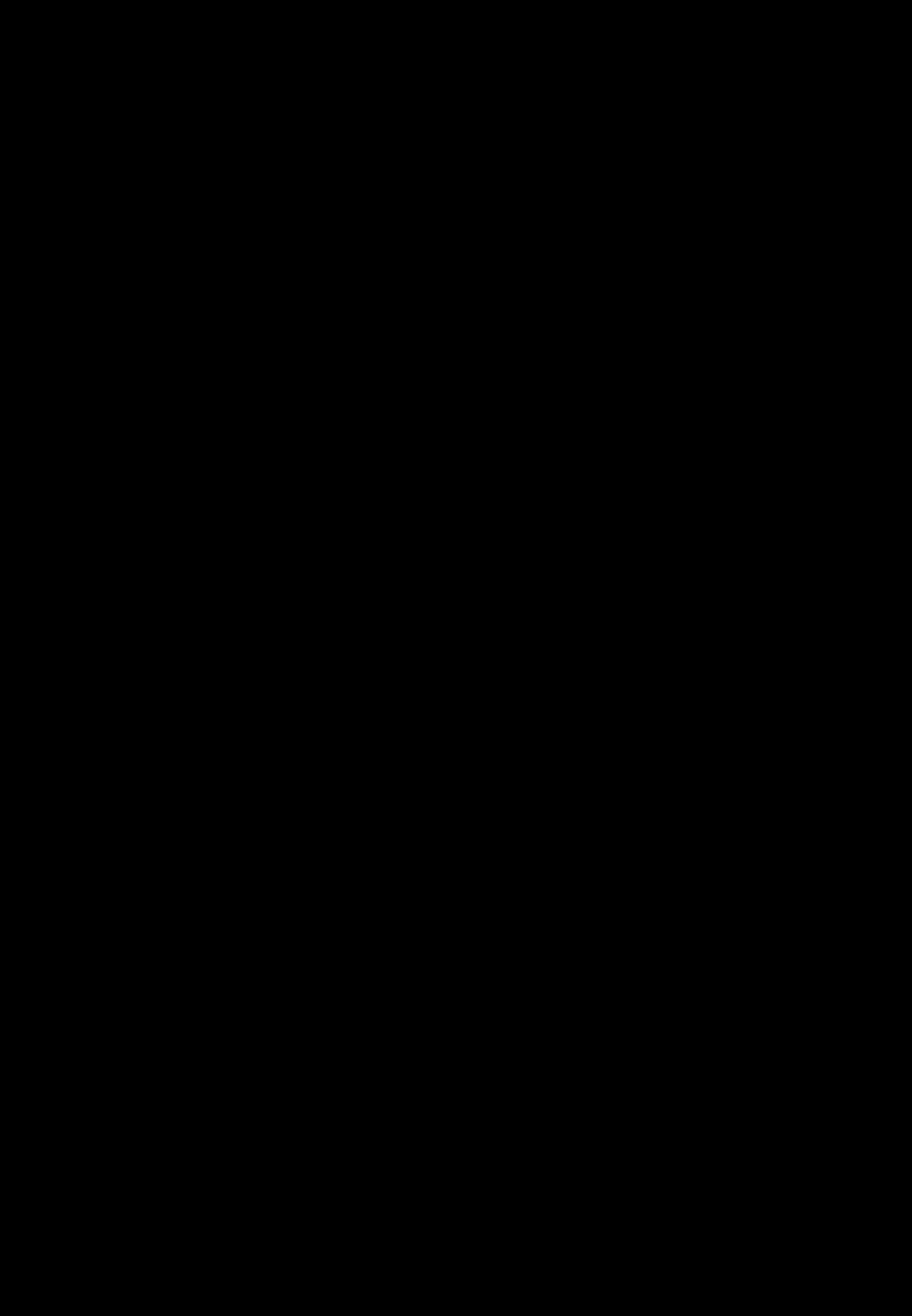

Supplement: Figure S1 — Membrane array analysis of intracellular signaling kinases affected by ATG conditioned PBMC supernatants in human cardiac myocytes. As shown, p53 was down-regulated after exposure to conditioned supernatants obtained from ATG stimulated PBMC cultures. (TIF) [file pone.0052101.s001.tif]
