# Supplementary material for: Anti-Thymocyte Globulin Induces Neoangiogenesis and Preserves Cardiac Function after Experimental Myocardial Infarction
Source: PLoS One. 2012 Dec 20;7(12):e52101. doi: 10.1371/journal.pone.0052101 (PMC3527351; doi:10.1371/journal.pone.0052101)
Supplement: Table S1 — Membrane array coordinates are shown for intracellular signaling kinases affected by ATG conditioned PBMC supernatants in human cardiac myocytes (see Figure S1). (DOC) [file pone.0052101.s002.doc]

**Table S1**

| A1,2:  pos con | B1,2:  --- | C1,2:  TOR | D1,2:  Src | E1,2:  Fyn | F1,2:  Hck | G1,2:  Pos con |
| --- | --- | --- | --- | --- | --- | --- |
| A3,4:  p38α | B3,4:  MEK1/2 | C3,4:  CREB | D3,4:  Lyn | E3,4:  Yes | F3,4:  Chk-2 | G3,4:  --- |
| A5,6:  ERK1/2 | B5,6:  MSK1/2 | C5,6:  HSP27 | D5,6:  Lck | E5,6:  Fgr | F5,6:  FAK | G5,6:  Neg con |
| A7,8:  JNK pan | B7,8:  AMPKα1 | C7,8:  AMPKα2 | D7,8:  STAT2 | E7,8:  STAT3 | F7,8:  STAT6 | G7,8:  --- |
| A9,10:  GSK-3α/β | B9,10:  Akt | C9,10:  β-Catenin | D9,10:  STAT5a | E9,10:  STAT5b | F9,10:  STAT5a/b | G9,10:  --- |
| A11,12:  --- | B11,12:  Akt | C11,12:  p70 S6 Kinase | D11,12:  p70 S6 Kinase | E11,12:  p70 S6 Kinase | F11,12:  STAT1 | G11,12:  --- |
| A13,14:  p53 (S392) | B13,14:  p53 (S46) | C13,14:  p53 (S15) | D13,14:  RSK1/2/3 | E13,14:  RSK1/2 | F13,14:  STAT4 | G13,14:  --- |
| A15,16:  --- | B15-16:  --- | C15,16:  p27 | D15,16:  p27 | E15,16:  c-Jun | F15,16:  eNOS | G15,16:  --- |
| A17,18:  pos con | B17,18:  --- | C17,18:  Paxillin | D17,18:  PLCγ-1 | E17,18:  Pyk2 | F17,18:  neg con | G17,18:  --- |
